# Multidrug Resistance 1 Gene Variants, Pesticide Exposure, and Increased Risk of DNA Damage

**DOI:** 10.1155/2014/965729

**Published:** 2014-03-26

**Authors:** Chun-Chieh Chen, Chun-Huang Huang, Man-Tzu Marcie Wu, Chia-Hsuan Chou, Chia-Chen Huang, Tzu-Yen Tseng, Fang-Yu Chang, Ying-Ti Li, Chun-Cheng Tsai, Tsung-Shing Wang, Ruey-Hong Wong

**Affiliations:** ^1^Department of Family and Community Medicine, Chung Shan Medical University Hospital, Taichung 40201, Taiwan; ^2^Department of Occupational Medicine, Chung Shan Medical University Hospital, Taichung 40201, Taiwan; ^3^School of Medicine, Chung Shan Medical University, Taichung 40201, Taiwan; ^4^Department of Public Health, Chung Shan Medical University, Taichung 40201, Taiwan; ^5^Department of Clinical Pharmacy, School of Pharmacy, Taipei Medical University, Taipei 110, Taiwan; ^6^Department of Pharmacy, Taipei Medical University, Wan Fang Hospital, Taipei 110, Taiwan; ^7^School of Biomedical Sciences, Chung Shan Medical University, Taichung 40201, Taiwan

## Abstract

The P-glycoprotein, encoded by the multidrug resistance (*MDR*)*1* gene, extrudes fat-soluble compounds to the extracellular environment. However, the DNA damage of pesticides in subjects with genetic variation in* MDR1* has not been investigated. In this study, the comet assay was applied to examine the extent of DNA damage in the peripheral blood of 195 fruit growers who had been exposed to pesticides and 141 unexposed controls. The *MDR1* polymorphisms were identified. Questionnaires were administered to obtain demographic data and occupational history. Results showed subjects experiencing high (2.14 *μ*m/cell, *P* < 0.01) or low pesticide exposure (2.18 *μ*m/cell, *P* < 0.01) had a significantly greater DNA tail moment than controls (1.28 *μ*m/cell). Compared to the *MDR1* T-129C (rs3213619) TC/CC carriers, the TT carriers had increased DNA tail moment in controls (1.30 versus 1.12 *μ*m/cell, *P* < 0.01). Similar results were observed in the high and low pesticide-exposed groups. Combined analysis revealed that pesticide-exposed fruit growers with *MDR1* -129 TT genotype had the greatest DNA damage in the subjects with the combinations of pesticide exposure and *MDR1* -129 genotypes. In conclusion, pesticide exposed individuals with susceptible *MDR1* -129 genotypes may experience increased risk of DNA damage.

## 1. Introduction

Although pesticide exposure has been linked to an increased risk of many cancers [1–4], epidemiologic data on the cytogenetic effects in pesticide-exposed farmers have been inconsistent [5–7]. Previously, our studies in Taiwan reported that pesticide-exposed fruit growers had a higher risk of DNA damage [8, 9]. In particular, genetic susceptibility has a substantial contribution to DNA damage in pesticide-exposed population.

The P-glycoprotein (P-gp) is encoded by the human multidrug resistance protein 1 (*MDR1* or* ABCB1*) gene. Importantly, this protein can extrude lipophilic compounds to the extracellular space by the ATP-dependent efflux transport mechanism, including chemotherapeutic agents and pesticides [10, 11]. It has also been reported that P-gp acts as an epithelial barrier and performs as excretory functions in various normal human tissues [11]. An animal study found that constructed* mdr1a*-disrupted mice which compared to normal* mdr1a* mice had the increased toxicity by the pesticide, and there was decreased elimination of this compound [12]. In particular, P-gp is capable of interacting with a large group of structurally diverse pesticides [13]. Therefore, P-gp might play a critical role in the detoxification of pesticide.

Alterations in P-gp expression and function potentially depend on structural variations of the* MDR1* gene. Human* MDR1* is located on chromosome 7q21.1, and many single nucleotide polymorphisms (SNPs) within this gene have been identified. The two common synonymous SNPs are C3435T (rs1045642), located in exon 26 at position 3435, and C1236T (rs1128503), located in exon 12 at position 1236 [14]. It has been found that the mRNA expression and P-gp activity of 3435T and 1236T alleles each were significantly lower than those of the 3435C and 1236C alleles [15–17]. The other frequent nonsynonymous SNP is G2677T/A (rs2032582), which is located in exon 22 at position 2677. This polymorphism could change the amino acid from alanine (*Ala*) to Serine (*Ser*) or threonine (*Thr*) and result in the lower P-gp expression [15]. A previous study conducted in Chinese subjects observed that the* MDR1* C3435T, C1236T, and G2677T/A genetic polymorphisms were significantly associated with a higher risk of developing Parkinson's disease [18]. These three polymorphisms were also indicated to be closely related to linkage disequilibrium. Thus, the haplotypes composed of different* MDR1* SNPs might have a better representation of a change in P-gp function [19, 20]. Another important* MDR1* SNP is T-129C (rs3213619). This polymorphism is located in the promoter region, and it has been established that -129C allele has a decreased P-gp expression [21].

Although the role of* MDR1* polymorphisms, particularly C3435T, C1236T, G2677T/A, and T-129C, has been evaluated in previous studies [22–24], little is known about their potential effect on the genotoxicity of pesticide. In this study, we investigated the association of these four* MDR1 *polymorphisms in pesticide-exposed fruit growers with cellular DNA damage, as measured by the comet assay.

## 2. Materials and Methods

### 2.1. Study Population and Epidemiological Information

The study design and final report were approved by the institutional review board of the Chung Shan Medical University, Taichung, Taiwan. All participants were provided with a written description of the study. Those who were unable to read the description had it read to them. All subjects gave written informed consent prior to inclusion in the study.

Previously, we conducted a cross-sectional study to explore the association between DNA damage and metabolic and DNA repair traits among 135 pesticide-exposed fruit growers and 106 nonexposed controls in Tungshin Town, which is located in central Taiwan. Criteria for selection of the study population are described in detail elsewhere [9]. In order to acquire greater statistical power to detect a difference in the level of DNA damage, sample size in the current study was increased to 195 pesticide-exposed fruit growers and 141 nonexposed controls. Fruit growers who were exposed to pesticides and unexposed controls were invited to participate in our study. The occupations of control subjects included housewives, teachers, clerks, nonfarm laborers, skilled workers, small-business persons, and professionals. We tried to minimize biases due to variations in ethnicity and lifestyle by selecting unexposed controls who were from the same residential area and of the same ethnicity as the pesticide-exposed subjects. None of the study subjects had received any therapeutic irradiation, and none were taking any medication.

A questionnaire on demographic characteristics, smoking, alcohol drinking, and occupational and medical histories was completed by each subject. The number of cigarettes smoked daily and the duration of the subject's smoking habit were also noted. Most of these farmers have been alerted to the risk of alcohol induced liver damage and understand that drinking alcohol makes the effects of pesticide poisoning worse. In general, alcohol drinking during the period of pesticide application is not allowed. We are concerned that if pesticide-exposed subjects with this condition were included, they would have a lower rate of alcohol drinking than the controls. Therefore, subjects who drank alcohol were not included in this study.

### 2.2. Assessment of Pesticide Exposure

The assessment of pesticide exposure has been described previously [8, 9, 25]. On the farms of our study area, pesticides are regularly applied all year. Information on past pesticide use by name, amount, area of pesticide application, numbers of treatments per season, years of agrochemical exposure, and use of personal protection equipment was obtained via interviewer-administered questionnaires in this study. Types of work in the orchards were also obtained. The pesticides used by the fruit growers during the 6 months before the medical examination consisted of almost 40 different compounds. On average, each farmer had applied pesticide about 3 times a month, with an average cumulative spraying duration of about 7 h/month (range, 2–28 h/month). Because of the lack of environmental monitoring data and the degree of personal protection used during handing pesticides, it is difficult to reconstruct an individual's previous pesticide exposure history. Thus, fruit growers were categorized as having low or high pesticide exposure by a modification of the criteria developed by Scarpato et al. [26]: (a) for each subject spraying pesticides, the number of hectares treated was determined, and pesticide exposure was calculated by multiplying the average number of treatments by the number of hectares sprayed; (b) the median value of the distribution obtained in (a) was determined, and fruit growers with exposure values less than or greater than the median were assigned to the low or high exposure class, respectively; and (c) subjects who did not directly handle pesticides (e.g., only involved in cutting or harvesting fruit) were considered to have low exposure. There was a good correlation between individuals' long-term exposure as estimated by our exposure model and acetylcholine esterase level. Thus, our estimation for pesticide exposure in this study should be acceptable.

### 2.3. Comet Capture and Analysis

In the present investigation, blood samples were collected in a single season (March-May), and each fruit grower was sampled at the beginning of a midweek working day. Blood samples from the study subjects were collected in heparinized tubes. The comet assay was conducted under alkali conditions according to Singh et al. [27]. For each subject, 100 randomly captured comets from slides (25 cells on each of four comet slides) were examined at ×400 magnification using an epifluorescence microscope connected through a black and white camera to an image analysis system (Comet Assay II; Perceptive Instruments Ltd., Haverhill, Suffolk, United Kingdom). Images acquired by the computerized image analysis system were used to compute the integrated intensity profiles for each cell, estimate the comet cell components, and evaluate the range of derived parameters. To quantify DNA damage, the tail moment was calculated as the product of the tail length and the fraction of DNA in the comet tail. A single reader, who was blind to the status of subjects, scored all slides.

### 2.4. Genotyping of Polymorphic MDR1 Genes

Genomic DNA was extracted from peripheral blood using the AxyPrepTM Blood Genomic DNA Miniprep Kit (Axygen Scientific, Union City, CA, USA).* MDR1* C3435T (rs1045642) polymorphism was analyzed by polymerase chain reaction (PCR)- based restriction fragment length polymorphisms [16]. Primers used for the amplification of the rs1045642 were 5′-TGC TGG TCC TGA AGT TGA TCT GTG AAC-3′ and 5′-ACA TTA GGC AGT GAC TCG ATG AAG GCA-3′. PCR products were digested with* Dpn*II.* MDR1* C1236T (rs1128503), G2677T/A (rs2032582), and T-129C (rs3213619) polymorphisms were determined by the StepOne Real-Time PCR System (Applied Biosystems) and analyzed by SDS v3.0 software (Applied Biosystems), using the TaqMan assay (assay IDs: C_7586662_10 for rs1128503, C-11711720C_30 for rs2032582 A/C, C-11711720D_40 for rs2032582 C/T, and C_27487486_10 for rs3213619) [28]. Approximately 10% of the randomly selected samples were directly sequenced to examine the initial genotyping results.

### 2.5. Statistical Analysis

The continuous variables were presented by mean ± standard error (SE) and were compared by Student's *t*-test and ANOVA among different pesticide exposure groups and control groups. The categorical variables among different pesticide exposure groups and controls were presented by numbers (%) and were compared by *χ* ^2^-test or Fisher's exact test. Hardy-Weinberg equilibrium was performed to test* MDR1* genotypes for goodness of fit. Subsequently, the crude DNA tail moment was evaluated using an analysis stratified by pesticide exposure and different factors. ANOVA was used to compare difference in DNA tail moment by different pesticide exposure groups and control groups, and Student's *t*-test or ANOVA was used to test the association of the DNA tail moment with age, gender, smoking status, and* MDR1* genotypes. Linkage disequilibrium (LD) coefficients, *D*′ = *D*/*D*_max⁡_ (or *D*/*D*_min⁡_ if the *D*′ value is negative), were assessed for pairs of alleles between* MDR1* rs1045642, rs3213619, rs1128503, and rs2032582 polymorphisms by the expectation-maximization algorithm. We estimated the common haplotypes by the expectation-maximization algorithm. Differences in DNA tail moment among different haplotypes were evaluated by ANOVA in the different pesticide exposure groups and controls, respectively. Further, the association of pesticide exposure and* MDR1 *genotypes with the DNA tail moment was analyzed using a general linear model (GLM) and adjusting the effects of confounding factors. In addition, least squares means were calculated to predict adjusted DNA tail moment for study subjects stratified by pesticide exposure status and genotypes; and tests for differences in least squares means were also performed. All *P* values were calculated using two-tailed statistical tests, and statistical significance was defined at *P* < 0.05. All data were analyzed using SAS 9.1 software (SAS Institute, Cary, NC, USA).

## 3. Results

Basic characteristics of pesticide-exposed fruit growers and controls are presented in Table 1. The control group was significantly younger (*P* < 0.01, ANOVA) and with a lower proportion of males (*P* < 0.01, *χ*2-test) compared to the high and low pesticide-exposed groups. The control group also had fewer pack-years of smoking than the pesticide-exposed groups (*P* < 0.01). In addition, the mean size of the orchards differed significantly between the high and low pesticide-exposed groups (*P* < 0.01, *t*-test). The prevalence of* MDR1 *genotypes among the study subjects is shown in Table 2. In all subjects, the* MDR1* C3435T (rs1045642, *P* = 0.08) and C1236T (rs1128503, *P* = 0.36) genotypes conformed to the Hardy-Weinberg equilibrium, whereas the G2677T/A (rs2032582) and T-129C (rs3213619) genetic polymorphism did not (*P*s < 0.001). The prevalence of* MDR1* C3435T, C1236T, G2677T/A, and T-129C polymorphisms among the different pesticide exposure and control groups was not significantly different.

The crude associations of DNA tail moment with various factors are presented in Table 3. Subjects in the low (2.18 *μ*m/cell, *P* < 0.001) and high (2.14 *μ*m/cell, *P* < 0.001) pesticide-exposed groups had higher DNA tail moment than controls (1.28 *μ*m/cell), respectively. In the control group, subjects younger than 53 years (mean age of all subjects), males, and those who smoked more than 10 pack-years also showed higher DNA tail moment than those older than 53 years (1.31 versus 1.20 *μ*m/cell, *P* < 0.01), females (1.34 versus 1.22 *μ*m/cell, *P* < 0.01), and those who smoked less than 10 pack-years (1.39 versus 1.26 *μ*m/cell, *P* < 0.01), respectively. Interestingly, the DNA tail moment was found to be significantly higher for control subjects with the* MDR1*-129 TT genotype than that of subjects with TC or CC genotypes (1.30 versus 1.12, 1.11 *μ*m/cell, *P* < 0.01; ANOVA). Since the expression of P-gp is lower in subjects with the* MDR1* -129C allele than subjects with the* MDR1*-129T allele [21], thus those with* MDR1*-129 TC and CC genotypes were further combined for the analysis. Significant difference in the DNA tail moments still remained between the groups of those with* MDR1*-129 TT and TC/CC genotypes (1.30 versus 1.12 *μ*m/cell, *P* < 0.01). However, the DNA tail moment was not associated with the* MDR1* C3435T, C1236T, and G2677T/A genotypes. Similar results were observed in the high and low pesticide-exposed groups.

**Figure 1:**
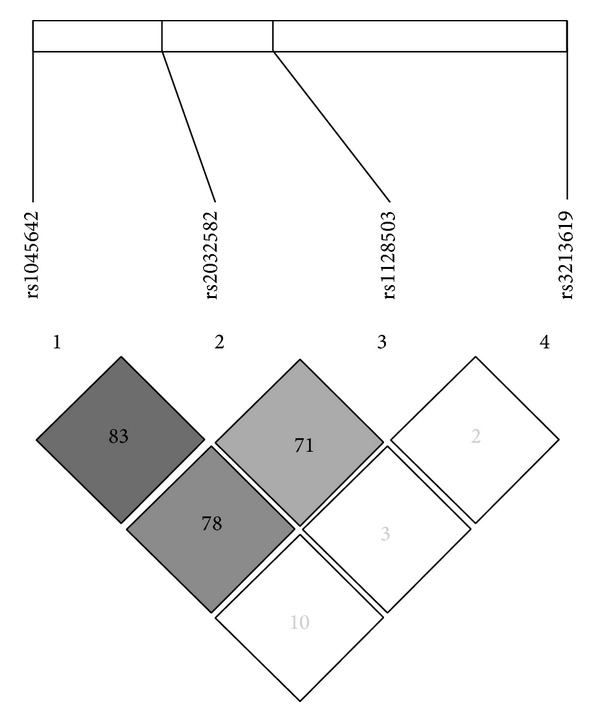
Linkage disequilibrium (LD) and haplotype block structure of* MDR1* gene. Numbers in squares represent the pairwise* D*′ value.

Furthermore, haplotype analysis using the expectation-maximization algorithm showed that the rs1045642, rs1128503, and rs2032582 are in tight linkage disequilibrium with each other (*D*′ value of >0.7, Figure 1) but the rs3213619 is not in linkage disequilibrium with the former ones. Therefore, the haplotype determination was limited to rs1045642, rs2032582, and rs1128503. The average DNA tail moments per cell stratified by* MDR1* C3435T, C1236T, and G2677T/A haplotypes are presented in Table 4. Among the 12 possible haplotypes, TTC (30.1%), CGC (24.6%), and CGT (19.5%) were predominant in all study subjects. The average DNA tail moments per cell in these haplotypes were not significantly different among high and low pesticide-exposed groups and controls.

**Figure 2:**
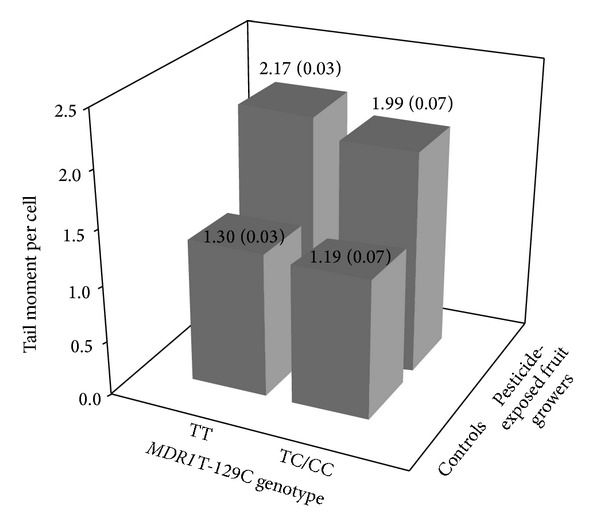
DNA tail moment per cell stratified by the* MDR1* T-129C (rs3213619) genotypes and pesticide exposure (standard errors in parentheses). Least squares mean analysis was performed to adjust for the effects of age and gender.

A multiple linear regression model for the relationship between DNA tail moment and age, gender, smoking status, pesticide exposure, and genotypes of* MDR1 *T-129C is shown in Table 5. The DNA tail moment was significantly and positively associated with males, high pesticide exposure, low pesticide exposure, and* MDR1*-129 TT genotype (*P*s < 0.01). Subsequently, a least squares mean analysis was performed to assess the joint effect of the* MDR1* T-129C polymorphisms and pesticide exposure on DNA tail moment after adjusting for the confounding effects (Figure 2). As statistical power was considered, the low and high pesticide exposure groups were combined. Controls with* MDR1*-129 TC/CC genotypes were selected as the referent group. Compared to the referent group (1.19 ± 0.05 *μ*m/cell, *n* = 21), pesticide-exposed fruit growers with* MDR1*-129 TT had significantly higher tail moment (2.17 ± 0.03 *μ*m/cell, *n* = 174, *P* < 0.01), followed by pesticide-exposed fruit growers with* MDR1*-129 TC/CC genotypes (1.99 ± 0.07 *μ*m/cell, *n* = 21, *P* < 0.01) and controls with* MDR1*-129 TT genotype (1.30 ± 0.03 *μ*m/cell, *n* = 120, *P* = 0.02).

## 4. Discussion 

It is important to identify the potential susceptibility factors affecting individual genotoxicity in response to pesticide exposure. In the present study, we investigate the association of pesticide exposure and cellular DNA damage, as measured by the comet assay, which is a sensitive method of assessing DNA damage. The comet assay of peripheral blood samples in our study and several previous studies has revealed greater DNA damage in individuals who had been exposed to complex mixtures of pesticides [29, 30]. In our previous studies, genetic variability in the enzymes that metabolize agricultural chemicals or repair DNA damage was also observed to be involved in the genotoxic process in response to pesticide exposure [8, 9]. In the present study, we observed that* MDR1*-129 TT genotype carriers had significantly higher DNA tail moment than TC/CC genotypes carriers. Further, pesticide-exposed fruit growers with* MDR1*-129 TT genotype had the greatest DNA damage in subjects with combinations of pesticide exposure and* MDR1* C-129T genotypes.

DNA damage can be induced by environmental carcinogens like pesticides and/or through metabolic or poor DNA repair processes that increase genomic instability [8, 9]. In addition to metabolic and DNA repair genes, transmembrane transporters on the surface of cells also may have an important role in the protection against gene instability and cancer initiation induced by long-term pesticide exposure. The P-glycoprotein, encoded by* MDR1 *gene, is an efflux pump to minimize exposure to chemicals by removing compounds from cells in mammals [10, 11]. It has been found that several* MDR1 *genetic polymorphisms are related to a functional variation of the protein [15–17, 21]. Therefore, there is a strong rationale for exploring the role of* MDR1* polymorphisms in genetic susceptibility to DNA damage among pesticide-exposed fruit growers in our current study.

In the current study, it was found that the* MDR1* C3435T and C1236T genotypes conformed to the Hardy-Weinberg equilibrium, while the* MDR1* G2677T/A and T-129C polymorphisms did not conform. However, reports in the NCBI Variation Database indicate that both the G2677T/A and the T-129C polymorphisms from persons of Chinese descent (HAPMAP CHB) also did not conform to the Hardy-Weinberg equilibrium. In addition, the frequency of the* MDR1* 3435T allele (37.2%) in our subjects is consistent with the result of HAPMAP CHB report (38.7%). The prevalence of the* MDR1* 1236T allele (64.6%) in our study also appears to be quite similar to that previously reported for ethnic Chinese (63.8%) [31]. The frequencies of the* MDR1 *2677 GG genotype (26.5%) and G allele (47.0%) from our subjects were similar to those reported from HAPMAP CHB (GG genotype: 26.8%; G allele: 43.9%). The prevalence of the* MDR1*-129C (7.7%) in our study subjects was close to that reported from HAPMAP CHB (6.1%). These findings, to some extent, validate the practice and results of our genotyping technique. In addition, the current study recruited 195 pesticide-exposed fruit growers and 141 nonexposed controls. Given a type I error (*α*) level of 0.05, the numbers of our subjects with* MDR1* T-129C TT genotype and those with TC/CC genotypes were 294 and 42, respectively; and the detectable difference of average tail moment between the subjects carrying* MDR1* T-129C TT genotype (mean (SE): 1.82 ± 0.03) and those carrying TC/CC genotypes (1.55 ± 0.08) was 0.27. We acquired a sufficient statistical power of 0.88.

It has been proposed that P-gp is capable of interacting with a large group of structurally diverse pesticides [13]. Interestingly, we observed that* MDR1*-129 TT genotype carriers had the significantly higher DNA tail moment than TC/CC genotypes carriers. The* MDR1 *T-129C polymorphism is located in the promoter region, 7 bp downstream from the transcription initiation site. The* MDR1*-129C allele has also been reported to have a lower P-gp expression than the -129T allele [21]. From the combined analysis, we further observed that pesticide-exposure fruit growers with* MDR1* -129 TT genotype had the greatest DNA tail moment, followed by pesticide-exposure fruit growers with* MDR1* -129 TC/CC genotypes and controls with* MDR1* -129 TT genotype. The DNA tail moment of controls with* MDR1* -129 TC/CC genotypes was significantly smaller than those of other groups of combined pesticide exposure and* MDR1* -129 genotypes. Thus, our findings suggest that the* MDR1* T-129C polymorphism may modulate susceptibility to the genotoxicity of pesticides. To the best of our knowledge, this is the first study on the association of* MDR1* genetic polymorphisms in pesticide-exposed fruit growers with cellular DNA damage. However, our results need to be replicated in other populations since it is likely that the* MDR1* T-129C polymorphism may be a susceptibility factor for genotoxicity of pesticides only in certain ethnic groups.

A previous study observed that the* MDR1* C3435T, C1236T, and G2677T/A genetic polymorphisms were significantly associated with a higher risk of developing Parkinson's disease in Chinese subjects [18]. However, these SNPs were not significantly associated with childhood acute lymphoblastic leukemia [32]. In the present study, the DNA tail moment was also not associated with the* MDR1* C3435T, C1236T, and G2677T/A genotypes. Further, the role of* MDR1* C1236T-G2677T/A-C3435T haplotypes has also been examined and provided evidence of a differential effect of indoor insecticide exposure on acute lymphoblastic leukemia risk in children with different haplotypes [32]. As expected,* MDR1 *C3435T, C1236T, and G2677T/A were in tight linkage disequilibrium with each other, but the* MDR1* -129 was not in linkage disequilibrium with the former ones in our haplotype analysis. However, differences in the average DNA tail moment per cell stratified by* MDR1* haplotypes did not reach statistical significance among high and low pesticide-exposed groups and controls. Since some genetic polymorphisms may exert population-specific effect, the “at-risk” allele in one person may not be an “at-risk” allele in another. Therefore, the lack of any association of the DNA tail moment with the* MDR1* C3435T, C1236T, and G2677T/A individual genotype or haplotype in our study may partly be due to different environmental exposure and different study populations.

In our study area, most of the younger residents have a low regard for agricultural work. Thus, the agricultural population tends to be older, and our control group was significantly younger than pesticide exposure groups. As expected, the older farmers who smoked also had more pack-years of smoking than younger farmers. Although adjustment was also performed for the confounding factors such as age, gender, and smoking status in our multiple regression model, the effect of selection bias might remain. The present study showed that smoking was not associated with DNA tail moment, which is probably because fewer cigarettes were smoked by subjects in the current study than in other studies [33]. In addition, nondifferential misclassification of pesticide exposure in the current study is likely to occur and, if apparent, can lead to an underestimation of the risk of DNA damage. Furthermore, data pertaining to individual exposure were obtained without the knowledge of health outcome. Lastly, it is not surprising that the P-gp activity phenotypes will provide additional information about the risk of DNA damage in pesticide-exposed subjects that was not provided by genotype alone from our study.

In conclusion, the results reveal that individuals with susceptible* MDR1* -129 genotypes may experience an increased risk of DNA damage due to pesticide exposure.

## Figures and Tables

**Table 1:** Basic characteristics of pesticide-exposed fruit growers and controls.

Variables	Controls	Pesticide exposure	
		Low
Number of subjects	141	82	113
Age (years)	49.0 ± 0.9*	55.5 ± 1.2	54.7 ± 1.1
Gender: male (%)	68 (48.2%)*	47 (57.3%)	84 (74.3%)
Duration of pesticide exposure (years)	0	29.9 ± 1.7	30.1 ± 1.4
Size of orchard (ha)	0	0.8 ± 0.1	1.7 ± 0.1^#^
Smoking habit			
Currents smoker (%)	26 (18.4%)*	23 (28.0%)	33 (29.2%)
Pack-years	3.7 ± 0.8*	8.2 ± 1.7	9.4 ± 1.5

Data represent numbers of individuals or mean ± SE for continuous variables.

*P < 0.01; control group differed significantly from the high and low pesticide-exposed groups.

^#^ *P* < 0.01 compared with the low pesticide-exposed group.

**Table 2:** Prevalence of *MDR1 *genotypes among pesticide-exposed fruit growers and controls.

*MDR1 *genotypes		Controls	Pesticide exposure		*P* value
			Low	High	
Number of subjects		141	82	113	
C3435T	CC	64 (45.4%)	29 (35.4%)	47 (41.6%)	0.62
(rs1045642)	CT	56 (39.7%)	40 (48.8%)	46 (40.7%)	
	TT	21 (14.9%)	13 (15.8%)	20 (17.7%)	
C1236T	CC	22 (15.6%)	12 (14.6%)	12 (10.6%)	0.71
(rs1128503)	CT	59 (41.8%)	33 (40.3%)	54 (47.8%)	
	TT	60 (42.6%)	37 (45.1%)	47 (41.6%)	
G2677T/A	GG	36 (25.5%)	23 (28.1%)	30 (26.5%)	0.78
(rs2032582)	GT/GA	54 (38.3%)	36 (43.9%)	48 (42.5%)	
	TA/TT/AA	51 (36.2%)	23 (28.0%)	35 (31.0%)	
	Non-GG	105 (74.5%)	59 (71.9%)	83 (73.5%)	0.92
T-129C	TT	120 (85.1%)	72 (87.8%)	102 (90.3%)	0.72*
(rs3213619)	TC	15 (10.6%)	8 (9.8%)	9 (8.0%)	
	CC	6 (4.3%)	2 (2.4%)	2 (1.8%)	
	TC/CC	21 (14.9%)	10 (12.2%)	11 (9.7%)	0.46

*Frequencies of T-129C genotype among the groups of low and high pesticide exposure and controls were compared by Fisher exact test.

**Table 3:** Average tail moment per cell stratified by pesticide exposure status and various factors.

	Controls		Pesticide exposure			
Variables			Low		High	
	*n*	Mean ± SE	*n*	Mean ± SE	*n*	Mean ± SE
All	141	1.28 ± 0.01	82	2.18 ± 0.05*	113	2.14 ± 0.04*
Age (years)						
≥53	44	1.20 ± 0.02*	46	2.15 ± 0.07	60	2.17 ± 0.06
<53	97	1.31 ± 0.02	36	2.22 ± 0.08	53	2.11 ± 0.04
Gender						
Males	68	1.34 ± 0.02*	47	2.27 ± 0.08^#^	84	2.21 ± 0.04*
Females	73	1.22 ± 0.01	35	2.05 ± 0.06	29	1.95 ± 0.05
Smoking status						
>10 pack-years	19	1.39 ± 0.04*	21	2.30 ± 0.11	33	2.15 ± 0.07
≤10 pack-years	122	1.26 ± 0.01	61	2.14 ± 0.06	80	2.14 ± 0.04
*MDR1 *C3435T genotype						
CC	64	1.29 ± 0.02	29	2.02 ± 0.07	47	2.13 ± 0.05
CT	56	1.29 ± 0.02	40	2.26 ± 0.08	46	2.16 ± 0.06
TT	21	1.22 ± 0.03	13	2.27 ± 0.14	20	2.13 ± 0.08
*MDR1* C1236T genotype						
CC	22	1.31 ± 0.03	12	2.12 ± 0.15	12	2.06 ± 0.11
CT	59	1.29 ± 0.02	33	2.14 ± 0.08	54	2.19 ± 0.05
TT	60	1.25 ± 0.02	37	2.23 ± 0.08	47	2.11 ± 0.06
*MDR1* G2677T/A genotype						
GG	36	1.30 ± 0.03	23	2.22 ± 0.10	30	2.15 ± 0.07
GT/GA	54	1.25 ± 0.02	36	2.17 ± 0.08	48	2.12 ± 0.06
TA/TT/AA	51	1.29 ± 0.02	23	2.15 ± 0.09	35	2.16 ± 0.07
Non-GG	105	1.27 ± 0.01	60	2.16 ± 0.06	83	2.14 ± 0.04
*MDR1 *T-129C genotype						
TT	120	1.30 ± 0.01*	72	2.21 ± 0.06*	102	2.15 ± 0.04*
TC	15	1.12 ± 0.01	8	1.89 ± 0.09	9	2.07 ± 0.13
CC	6	1.11 ± 0.01	2	2.10 ± 0.03	2	1.92 ± 0.18
TC/CC	21	1.12 ± 0.01*	10	1.93 ± 0.08	11	2.04 ± 0.11

Comparisons among different pesticide-exposed status groups or (three) genotype groups conducted with ANOVA; comparisons between age, gender, smoking status, and (two) genotype groups conducted with *t*-test.

*P < 0.01.

^#^0.01 < *P* < 0.05.

**Table 4:** Average DNA tail moment per cell stratified by *MDR1 *haplotypes.

Haplotype			Controls		Pesticide exposure				All	
C3435T	G2677T/A	C1236T			Low		High			
(rs1045642)	(rs2032582)	(rs1128503)	n*	Mean ± SE	*n *	Mean ± SE	*n *	Mean ± SE	*n *	Mean ± SE
T	T	C	83	1.25 ± 0.02	51	2.24 ± 0.07	68	2.13 ± 0.05	202	1.79 ± 0.04
C	G	C	65	1.26 ± 0.02	43	2.19 ± 0.07	57	2.17 ± 0.05	165	1.81 ± 0.04
C	G	T	56	1.30 ± 0.02	34	2.16 ± 0.08	41	2.08 ± 0.06	131	1.77 ± 0.05
x	A	x^#^	43	1.31 ± 0.03	26	2.07 ± 0.09	37	2.18 ± 0.06	106	1.80 ± 0.05
Others^$^			35	1.30 ± 0.03	10	2.14 ± 0.19	23	2.17 ± 0.09	68	1.72 ± 0.07

*Number of alleles.

^#^Haplotypes contained the variant A allele at the G2677T/A locus (including CAT, CAC, TAT, and TAC).

^$^Rare haplotypes with frequencies <5% and not part of the variant A allele at the G2677T/A locus (including CTT, CTC, TGT, TGC, and TTT).

**Table 5:** Multiple regression model for tail moment per cell.

Variables	Regression coefficient	SE	*P* value
Intercept	1.20	0.10	<0.01
Age: per 1-year increment	−0.003	0.002	0.09
Gender: males versus females	0.19	0.04	<0.01
Smoking status: >10 versus ≤10 pack-years	−0.02	0.05	0.64
Pesticide exposure			
High versus control	0.83	0.04	<0.01
Low versus control	0.90	0.05	<0.01
*MDR1* T-129C (rs3213619) genotype			
TT versus TC/CC	0.15	0.05	<0.01
